# Novel evidence of CNV deletion in *KCTD13* related to the severity of isolated hypospadias in Chinese population

**DOI:** 10.3389/fped.2024.1409264

**Published:** 2024-09-10

**Authors:** Yijing Chen, Lijun Zhou, Fang Chen, Zhongzhong Chen, Yichen Huang, Yiqing Lv, Min Wu, Xiaoling Lin, Hua Xie

**Affiliations:** ^1^ Department of Urology, Shanghai Children’s Hospital, School of Medicine, Shanghai Jiao Tong University, Shanghai, China; ^2^ Clinical Research Center for Hypospadias, Pediatric College, School of Medicine, Shanghai JiaoTong University, Shanghai, China

**Keywords:** copy number variation, isolated hypospadias, Chinese children, *KCTD13*, severity

## Abstract

**Background:**

CNV in *KCTD13* has been identified to influence androgen receptor function via its changes in gene dosage, which might contribute to hypospadias. However, there is lack of population-level evidence to assess the contribution of *KCTD13* CNV to hypospadias.

**Methods:**

349 isolated hypospadias patients were recruited and their genotyping was performed using real-time qPCR. We use Database of Genomic Variants (DGV) and CNV calls from SNP-array intensity data in 1,008 Chinese healthy men as reference.

**Results:**

11.17% of patients were identified to have *KCTD13* CNV deletion, significantly higher than 0.05% in DGV (*P* < 0.001), but no cases found to have CNV duplication. Meanwhile, no CNV calls encompassing *KCTD13* region were detected in Chinese healthy men. Incidence of *KCTD13* CNV deletion was significantly increased with the severity of hypospadias, *P*__trend_ = 9.00 × 10^−6^. Compared to distal hypospadias, ORs for the proximal and midshaft were 10.07 (2.91–34.84) and 6.08 (1.69–21.84) respectively. In addition, the association between genital characteristics (stretched penile length and glans width) and *KCTD13* CNV showed no significance in hypospadias children (*P *> 0.05).

**Conclusions:**

We demonstrate *KCTD13* CNV deletion is strongly associated with hypospadias and its severity, but duplication is not, characterizing *KCTD13* genetic variation in more detail than previously described.

## Introduction

Hypospadias is one of genitourinary (GU) tract anomalies, characterized by abnormal urethral development during embryonic development, resulting in the opening of the urethra being located anywhere along the underside of the penis (1). Its prevalence is approximately 20.9 per 10,000 births and has been increasing over time (2). Based on the abnormal location of urethral meatus, hypospadias is classified into distal, midshaft and proximal types (1). It may occur as an isolated disorder or accompany with other malformations (1, 3). Genetic factors have been demonstrated to play a principal role in the etiology of hypospadias, accounting for 77% of the heritability (4). Despite monogenic and chromosomal causes of hypospadias account for about 30% of all cases, the majority of genetic factors remain unknown (5–7). Previous studies are mainly based on sequencing and genotyping, and research on structural variations in hypospadias is limited (8, 9). Although structural variations are known to contribute to the genetic etiology of birth defects (10), few validated genetic markers have been associated with the risk of hypospadias in the Chinese population (11).

Copy number variation (CNV), a form of structural variations, is often observed as submicroscopic duplications or deletions of large genomic fragments exceeding 1 kb in length (12). They contribute to the development of human diseases by disrupting coding sequences, interfering with long-term gene regulation, or altering gene dosage (12). In a cohort of disorders of sex sexual development (DSD), 17.6% (3/17) of isolated hypospadias patients were found to carry a certain proportion of submicroscopic CNV, with duplication and deletion events noted on 16p11.2 for hypospadias (13). However, the contribution of CNV to hypospadias is still unknown. Investigation of CNV in the development of isolated hypospadias may provide valuable supplementary information for genetic research on this condition.

A recent study identified CNV events in potassium channel tetramerization domain containing 13 (*KCTD13*) on 16p11.2, which has been suggested as a potential candidate gene for GU anomalies in American population (14). The study observed a significantly higher prevalence of *KCTD13* CNV among individuals with congenital anomalies of GU tract (2.58%) compared to the general population (0.10%) (14). However, due to the limited sample size of hypospadias cases in this study, the association between *KCTD13* CNV and isolated hypospadias remains unclear. Therefore, we hypothesize that *KCTD13* CNV may be identified in hypospadias in the Chinese population and could be associated with severity of hypospadias.

## Patients and methods

### Study cohort and design

In this study, we employed a case design (Table 1). A total of 349 patients with isolated hypospadias (no cryptorchidism and no micropenis) were recruited from Shanghai Children's Hospital of China between 2014 and 2021, ranging in age from 0.50 to 14.75 years (mean age: 2.98 ± 2.84 years; median age: 1.83 years). Clinical diagnosis was established by pediatric urologists and/or endocrinologists through direct clinical examination. Patients were excluded if they: (i) carried known karyotype abnormalities or abnormalities in the sex-determining region Y (*SRY*) gene abnormalities; (ii) were diagnosed with hypospadias as a result of disorders of sex development (DSD); (iii) showed endocrine disorder; (iv) were diagnosed with additional clinical features such as cryptorchidism or micropenis. According to the abnormal location of the urethral meatus, patients were classified into three categories: distal (glandular, coronal, and subcoronal: *n* = 129), midshaft (distal penile, midshaft, and proximal penile: *n* = 113), and proximal (penoscrotal, scrotal and perineal: *n* = 107). Stretched penile length (SPL) was standardized was measured by standardizing the dorsal length from the pubic bone to the apical end of the penis using a steel ruler after maximum traction (15), and a vernier caliper was used to measure the maximum width of the glans (16).

**Table 1:** General characteristics of the study population.

Characteristics^a^	Total	Types of hypospadias		
		Distal	Midshaft	Proximal
*N* (%)	349 (100.00)	129 (36.96)	113 (32.38)	107 (30.66)
Age, years	1.83 (1.21–3.29)	2.25 (1.29–4.67)	1.67 (1.21–3.75)	1.58 (1.17–2.67)
BMI, kg/m^2^b^^	15.97 (14.79–17.31)	15.84 (14.72–17.31)	16.02 (15.22–17.75)	15.63 (14.32–17.10)
SPL, cm^b^	5.00 (4.50–5.50)	5.05 (4.60–5.63)	5.20 (4.50–5.50)	4.80 (4.30–5.20)
Glans width, cm	1.00 (0.90–1.20)	1.10 (1.00–1.22)	1.10 (0.91–1.20)	1.00 (0.90–1.12)

^a^ Data were reported as median (IQR).

^b^ BMI, body mass index; SPL, stretched penile length.

All demographic and clinical information of participants were collected prior to surgery. This study was approved by the Ethics Committee of the Shanghai Children's Hospital in China (2022R045-E01), and written informed consents were obtained from all participants.

### DNA extraction

Peripheral blood was collected from all subjects in vacuum blood tubes containing EDTA-K2. Genomic DNA was extracted from peripheral blood samples using the Gentra Puregene Blood Kit (Qiagen, Dusseldorf, Germany). The concentration and purity of DNA were determined using a Nanodrop ND2000 UV–vis spectrophotometer (Thermo Fisher Scientific, USA), and genomic DNA was stored at −80°C prior to CNV detection.

### Determination of *KCTD13* CNV

All samples were genotyped using the same genotyping system at the Central Laboratory of Shanghai Children's Hospital. The Applied Biosystems protocols for the TaqMan quantitative real-time polymerase chain reaction (qPCR) method were used to assess CNV (*KCTD13*: Assay Hs01712568_cn) in each sample. TaqMan CNV reactions were performed in triplicate, and all the above reagents were obtained from Thermo Fisher Scientific (Thermo Fisher Scientific, Waltham, MA, USA). The overall concordance rate for the CNV of *KCTD13* among 10% of samples (39 duplicate samples) was 100%. The CNV assay was performed using an ABI QuantStudio6 Real-time PCR system. Same genotyping was analyzed using CopyCaller V2.1 software from Applied Biosystems.

### Generation of CNV calls from intensity data of the genome-wide genotyping

We generated CNV calls from a genome-wide dataset of healthy controls in Chinese Consortium for Prostate Cancer Genetics (ChinaPCa), including 1,008 male Han Chinese from the community population. Details on study characteristics were reported in our previous study (17). A total of 731,458 SNPs have been genotyped using Illumina Human OmniExpress BeadChips (Illumina, San Diego, California).

A standard quality control procedure was used for generating CNV calls. SNPs with the following conditions were excluded: (i) genotype call rate <90%; (ii) minor allele frequency (MAF) < 0.01; or (iii) *p* < 0.001 for the Hardy–Weinberg equilibrium (HWE) test. Log R ratio (LRR) and B allele frequency (BAF) were generated using GenomeStudio (San Diego, CA, USA) and CNVs were called using cnvPartition (San Diego, CA, USA). Samples with the following conditions were removed: (i) standard deviation (SD) of LRR > 0.3; (ii) BAF_drift value >0.01; (iii) waviness factor (WF) > 0.05 or WF < −0.05; (iv) samples with more than 50 CNV calls. After individual quality control, a total of 982 controls remained. Minimum number of probes of 5 and confidence score of 35 were set as threshold for CNV calling when using cnvPartition analysis.

### Statistical analysis

Mann-Whitney *U* test and *t*-test were utilized to investigate the statistical differences of quantitative variables with CNV. The Cochran-Armitage trend test was used to evaluate the trends of *KCTD13* CNV detection rates in patients with different hypospadias ranges. Logistic regression analysis was performed to evaluate the independent influencing factors of *KCTD13* CNV. A *p* value of <0.05 (two-sided) was considered to be statistical significant. All statistical analyses were performed using the Statistical Package for the Social Sciences (SPSS v.26.0, SPSS Inc.) and GraphPad Prism (v10.0.3, GraphPad Software). The Database of Genomic Variants (DGV), a database of human genomic structural variants (http://dgv.tcag.ca/), was used to as a reference for general population.

## Results

The study cohort comprised 349 hypospadias patients, with 129 (36.96%) having distal, 113 (32.38%) for midshaft, and 107 (30.66%) proximal hypospadias. Table 1 showed the demographic information, including age, body mass index (BMI), stretched penile length (SPL) and glans width in the study population. The median age of patients with hypospadias was 1.83 (1.21–3.29) years old, while BMI was 15.97 (14.79–17.31) kg/m^2^. Additionally, the median SPL and glans width were 5.00 (4.50–5.50) cm and 1.00 (0.90–1.20) cm, respectively.

**Figure 1:**
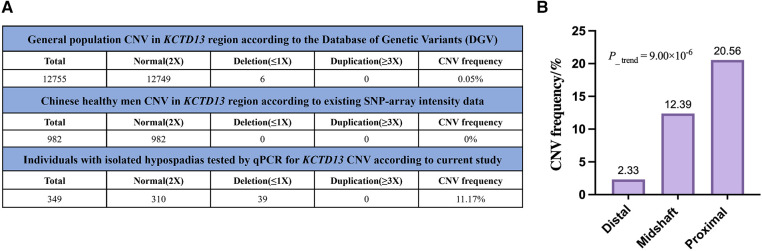
Patients with CNV in *KCTD13* gene at 16p11.2 exhibit genitourinary tract anomalies. (**A**) Comparison of the incidences of *KCTD13* CNV among our special disease cohort with isolated hypospadias, the general population from the Database of Genetic Variants (DGV) and Chinese healthy men according to existing SNP-array intensity data. (**B**) Distribution of *KCTD13* copy number deletion in patients with different severity of hypospadias. *P*__trend_ obtained using chi-squared tests of linear-by-linear association.

Table 2 showed the carried rates of CNV detection of *KCTD13* in the cohort. Among all patients, 11.17% (39 out of 349) were found to carry *KCTD13* CNV deletion (≤1), and none with CNV duplication (>2) in *KCTD13*. Chi-squared test analysis showed a statistically significant difference in the frequency of copy number deletion between the three groups (*P *= 4.90 × 10^−5^, Table 3), and there was an increasing trend in the rate of carrying copy number deletion of the gene as the severity of hypospadias increased (*P*__trend_ = 9.00 × 10^−6^, Figure 1).

**Table 2:** Frequency distributions of *KCTD13* CNV among different severity of hypospadias and its association with severity levels.

Types of hypospadias	Total	Deletion (≤1X)	Unadjusted		Adjusted^b^	
	*N*	*N* (%)	OR (95% CI)	*P*-value	OR (95% CI)	*P*-value
Total	349	39 (11.17)				
Proximal^a^	107	22 (20.56)	10.87 (3.15–37.46)	1.60 × 10^−4^	10.07 (2.91–34.84)	2.70 × 10^−4^
Midshaft^a^	113	14 (12.39)	5.94 (1.66–21.24)	0.006	6.08 (1.69–21.84)	0.006
Distal	129	3 (2.33)	1.00		1.00	

^a^ Reference group: Patients with distal hypospadias.

^b^ Adjustment for BMI.

**Table 3:** Comparison of general characteristics and clinical data of *KCTD13* CNV status among hypospadias patients.

Variables^a^	Copy number		
	Normal (2X)	Deletion (≤1X)	*P*-value^c^
Age, years	1.83 (1.17–3.25)	2.33 (1.33–4.83)	0.147
BMI, kg/m^2^^b,d^	15.99 (14.85–17.59)	15.38 (14.40–16.54)	0.031
SPL, cm^b,d^	5.00 (4.50–5.50)	5.00 (4.50–5.50)	0.650
Glans width, cm^b^	1.10 (0.90–1.20)	1.00 (0.90–1.18)	0.303
Classification, *N* (%)			
Distal	126 (40.65)	3 (7.69)	4.90 × 10^−5^
Midshaft	99 (31.94)	14 (35.90)	
Proximal	85 (27.34)	22 (56.41)	

^a^ Data were reported as median (IQR).

^b^ Number of subjects (*N*) was not equal to overall subjects.

^c^ *P*-values obtained using Mann-Whitney *U* test and chi-squared tests.

^d^ BMI, body mass index; SPL, stretched penile length.

Combining general population samples from a public database (Database of Genomic Variants, DGV), we found that the frequency of *KCTD13* CNV in the general population was low (0.05%, Figure 1). However, the samples in our study carried a much higher frequency of *KCTD13* CNV, up to 11.17%, which is significantly higher than the former (*P *< 0.001). Furthermore, cnvPartity-based analyses of our existing SNP-array intensity data didn't generate any CNV calls spanning *KCTD13* gene loci in the cohort of 1,008 healthy Chinese men.

Next, we performed the association of this CNV deletion with several clinical parameters. As shown in Table 3, the deletion was significantly correlated with lower levels of BMI (*P *= 0.031) and the more severe hypospadias (*P *< 0.001). However, the deletion was not related to SPL and Glans width, which are penis-related parameters. Further analysis showed that *KCTD13* CNV deletion was still significantly associated with more severe hypospadias after adjustment for BMI by logistic regression model (Table 2). It indicated that patients with *KCTD13* CNV deletion have a significantly increased risk for proximal hypospadias (OR = 10.07, 95% CI = 2.91–34.84, *P *= 2.70 × 10^−4^) and midshaft hypospadias (OR = 6.08, 95% CI = 1.69–21.84, *P *= 0.006) compared to the distal hypospadias.

Given the age, postnatal penile growth occurs concurrently with mini-puberty (from birth to 3 months of age) and then enters an extremely slow growth state before prepuberty (or during 1–3 years old). In our cohort, 62.18% (217/349) patients were aged between 1 and 3 years and 72.35% (157) of these had full record for SPL and glans width. Therefore, we selected these patients to reassess the correlation of *KCTD13* CNV with penile parameters. As the result, no correlation was found between SPL, glans width and *KCTD13* CNV in 1–3 years old children with isolated hypospadias (*P *> 0.05, Table 4).

**Table 4:** Penile parameters in 1–3 years old children with hypospadias.

Variables^a^	Total	Normal (2X)	Deletion (≤1X)	*P*-value^b^
*N* (%)	157 (100)	141 (89.81)	16 (10.19)	
Age, years	1.50 (1.25–2.17)	1.50 (1.17–2.15)	1.58 (1.33–2.33)	0.126
BMI, kg/m^2^^c^	15.97 (14.82–17.10)	15.97 (15.00–17.18)	14.79 (13.99–16.12)	0.007
SPL, cm^c^	5.00 (4.50–5.30)	5.00 (4.50–5.35)	4.90 (4.20–5.23)	0.485
Glans width, cm	1.05 ± 0.20	1.05 ± 0.20	1.02 ± 0.65	0.585

^a^ SPL data were reported as median (IQR), glans width data were reported as means ± SD.

^b^ *P*-values obtained using Mann-Whitney *U* test when abnormally distributed, two-sample *t*-test when normally distributed.

^c^ BMI, body mass index; SPL, stretched penile length.

## Discussion

In a previous study, *KCTD13* CNV with deletion and duplication was observed to be associated with clinical cases of upper and lower tract genitourinary (GU) anomalies, including more than hypospadias, in the American population, and its changes in gene dosage could result in penile and testicular anomalies via diminished androgen receptor (AR) function (14). However, the precise role of *KCTD13* CNV in hypospadias remains to be clarified. Our study determined the presence of *KCTD13* CNV in cases of hypospadias and contributed to the evidence, indicating that the deletion of CNV in *KCTD13* was significantly associated with increased risk to isolated hypospadias in a Chinese population. Although we did not find any association between *KCTD13* CNV and penile parameters in children aged 1–3 years with isolated hypospadias, the deletion of this CNV was positively correlated with the severity of hypospadias. To the best of our knowledge, this is the first reported study to demonstrate a relationship between *KCTD13* CNV and susceptibility to isolated hypospadias in the Chinese population.

In our study, the overall frequency of *KCTD13* CNV in hypospadias was 11.17%, which is much higher than that observed in patients with GU anomalies in the American population (2.58%) according to Seth et al. (14). Meanwhile, we utilized the existing SNP-array intensity data in our previous study (17) and the general population within the DGV database as the reference and found that the frequency of *KCTD13* CNV deletion in our cohort was also significantly higher than that in the general population (DGV: 0.05%), indicating its importance as a genetic marker for hypospadias susceptibility. Different from the previous research of Seth et al. (14), we strictly included isolated hypospadias as the study participants to rule out some unknown confounding variables, such as endocrine abnormality and symptom complex. All the detection of CNV type in *KCTD13* was deletion (single gene copy) and its carriage was more common among Chinese (11.17%) compared to the American population (14).

With our research, it added to the evidence different copy number of *KCTD13* reflected different contributions in the clinical phenotype. Previous research showed that a patient presenting both hypospadias and cryptorchidism exhibited *KCTD13* copy number duplications, whereas only one case with isolated hypospadias displayed *KCTD13* copy number deletion. In our study, all the determinations of *KCTD13* CNV type in isolated hypospadias patients were deletion. Our findings further validated these previous results and suggested that children with isolated hypospadias are more likely to exhibit *KCTD13* copy number deletion. The *KCTD13* gene is located at the 16p11.2 locus, and prior studies have demonstrated that 16p11.2 copy number deletions are more prevalent than duplications (18). These results reinforce the findings of the current study. On the other hand, deletion of copy number of *KCTD13* was also linked to other conditions like epilepsy and autism (19, 20). But limited research has been dedicated to investigate the association between CNV and the severity of diseases. In this study, we observed an association between *KCTD13* CNV and the severity of disease. Patients with distal, midshaft, and proximal isolated hypospadias all exhibited a certain proportion of *KCTD13* CNV, and the prevalence of *KCTD13* copy number deletion increased as the severity of hypospadias worsened (*P* < 0.001).

Some studies have demonstrated that the severity of hypospadias seemed to correlate indicators of masculinization, such as SPL and glans width. In an observational study, the research found severe hypospadias patients had significantly shorter SPL compared to mild one when evaluating actual post-pubertal penile size and factors affecting it in hypospadias patients (21). Similarly, Bush et al. observed a smaller glans width among patients with hypospadias aged 0–24 months than the boys presenting for newborn circumcision (16). Building upon findings from previous mouse models (14), *KCTD13*-deficient mice exhibit testicular and penile abnormalities at maturity. Consequently, our study delved further into the analysis of the correlation between penile parameters and *KCTD13* CNV. Considering the phenomenon of “small puberty” during infancy (from birth to 3 months), when the penis undergoes rapid growth (22), and the subsequent period of slow growth in children aged 1–3 years old (22–24), we selected patients within this age range for correlation analysis between penile parameters and *KCTD13* CNV. However, our study did not identify any significant associations between SPL or glans width and *KCTD13* CNV among isolated cases of hypospadias in children aged 1–3 years old. The limited number of samples available for comparison may have contributed to the absence of significant trends. The expansion of the sample in future studies may provide more clarity on the association between the aforementioned variables. Additionally, the previous study selected sexually mature *Kctd13^−/−^*mice for comparison of penile development (14). While our study excluded interference caused by age development, it is important to note that the majority of children have not yet reached sexual maturity. Therefore, assessing whether their penile development is affected by *KCTD13* CNV may not be appropriate, and long-term follow-up may be necessary to address this limitation.

*KCTD13* functions as a substrate-specific adapter of the BCR (BTB-CUL3-RBX1) E3 ubiquitin-protein ligase complex (14, 25) and plays integral roles in regulating cytoskeleton, migration, proliferation, and neural development (25–27). A study has found that knockdown of intracellular *KCTD13* reduces the translocation of AR into the nucleus upon androgen stimulation, which in turn affects the function of AR in transmitting androgen signals (14). Previous studies have indicated that the AR plays a crucial role in determining neonatal penile growth by controlling the proliferation of genital tubercle mesenchymal cells and promoting urethral fusion through the regulation of apoptosis. If these processes are compromised, it may result in the development of penile malformations like micropenis and hypospadias, and even lead to varying degrees of severity in hypospadias (28). However, abnormal expression, subcellular localization or function of AR can also contribute to hypospadias occurrence (28–30). Previous researches have demonstrated that E3 ubiquitin ligases can facilitate the degradation of AR (31, 32). The reduced expression of AR in testicular tissues of *Kctd13^−/−^* mice (14) raises the hypothesis that the deletion of *KCTD13* may lead to increased AR degradation, ultimately contributing to insufficient masculinization phenotypes. However, further studies are required to clarify the relationship between the two. Additionally, further research is still required to elucidate the molecular mechanisms underlying the association between *KCTD13* and hypospadias.

Up to date, there were few CNV studies particularly related to hypospadias. These studies predominantly included cases of hypospadias accompanied with other symptom and could only identified large piece of CNV (>1.5 Mb) (Supplementary Table) (3, 13), which may not fully represent the genetic etiology of isolated cases. Compared to large piece of CNV, smaller ones could specify candidate genes predisposing to the diseases and help bridge the gap in understanding complex genetic disorders like hypospadias. Therefore, the contribution of CNV to the etiology of isolated hypospadias remains largely unexplored. Hypospadias is a clinically and genetically heterogeneous disorder (7), it not only presents as a local deformity but also serves as an indication of systemic endocrine dysfunction, particularly proximal hypospadias, which in the majority of cases are ultimately due to problems with the child's sex hormone levels (33). A consensus thought that patients with isolated hypospadias were more likely to have normal endocrine testicular function by comparing testicular function between patients with isolated hypospadias and those with hypospadias with micropenis, cryptorchidism, or ambiguous genitalia (34). That's the reasons we chose the isolated hypospadias as a typical object of study for this project, which could exclude the potential interference of endocrine factors and phenotypic heterogeneity. Moreover, unlike previous studies, the sample size in our study was considerably large. And for the first time, we have demonstrated the high prevalence of *KCTD13* CNV among Chinese children with isolated hypospadias.

The limitation of this study lies in conducting only single gene copy number verification, without performing whole-genome CNV deletion analysis. Additionally, due to the majority of participants being young children with immature sexual development, there is a lack of data regarding penile development to comprehensively assess the degree of masculinization and predict its progression.

In the future, enhancing the long-term postoperative follow-up of children after surgery, particularly during puberty, will be beneficial for assessing and predicting the degree of masculinization. Furthermore, the prospective clinical utilization of *KCTD13* copy number deletion may aid in evaluating and predicting the long-term development of masculinization in children with varying degrees of hypospadias, especially those with proximal hypospadias.

## Conclusions

In conclusion, our study provides substantial evidence of an association between *KCTD13* CNV deletion and susceptibility to isolated hypospadias. This approach allows us to identify the genetic markers for isolated hypospadias in Chinese boys and to analyze differences in the genetic determinants of hypospadias between the Chinese population and other populations.

## Data Availability

The data presented in the study are deposited in the Figshare repository. The data is available here https://doi.org/10.6084/m9.figshare.26934262.v1
