# Correction: Novel evidence of CNV deletion in *KCTD13* related to the severity of isolated hypospadias in Chinese population

**DOI:** 10.3389/fped.2025.1753122

**Published:** 2025-12-18

**Authors:** Yijing Chen, Lijun Zhou, Fang Chen, Zhongzhong Chen, Yichen Huang, Yiqing Lv, Min Wu, Xiaoling Lin, Hua Xie

**Affiliations:** 1 Department of Urology, Shanghai Children’s Hospital, School of Medicine, Shanghai Jiao Tong University, Shanghai, China; 2 Clinical Research Center for Hypospadias, Pediatric College, School of Medicine, Shanghai JiaoTong University, Shanghai, China

**Keywords:** copy number variation, isolated hypospadias, Chinese children, *KCTD13*, severity

**Incorrect funding**

Missing

The funder [Shanghai Municipal Health Commission], [20224Y0323] to [Lijun Zhou] was erroneously omitted.

The original version of this article has been updated.

