# Supplementary material for: Novel evidence of CNV deletion in KCTD13 related to the severity of isolated hypospadias in Chinese population
Source: Front Pediatr. 2024 Sep 10;12:1409264. doi: 10.3389/fped.2024.1409264 (PMC11420791; doi:10.3389/fped.2024.1409264)
Supplement: Supplementary file 1 [file Table1.docx]

**Supplementary Table** Review of CNV involving hypospadias patients identified in previous studies.

| **No.** | **Authors** | **Published Year** | **Sample Size** | **Phenotype** | **CNV** | **PMID** |
| --- | --- | --- | --- | --- | --- | --- |
| 1 | Tannour-Louet, Mounia et al. | 2010 | 116 | Syndromic and non-syndromic congenital genitourinary disorders (such as hypospadias) | 12p13, 16p11.2 duplication and deletion | 21048976 |
|  |  |  |  | Syndromic and non-syndromic congenital genitourinary disorders (such as ambiguous genitalia) | 1p36.33, 9p24.3, 19q12-q13.11 duplication and deletion |  |
| 2 | Nelson, Marc et al. | 2011 | 1 | Multiple organ hypoplasia (including hypospadias) | 16p13.3 deletion | 21344629 |
| 3 | Gana, Simone et al. | 2012 | 2 | Multiple organ hypoplasia (including hypospadias) | 19q13.11 deletion | 22378287 |
| 4 | Tannour-Louet, Mounia et al. | 2014 | 116 | 46, XY disorders of sexual development (DSD) | Xq28 duplication | 24880616 |
| 5 | Kon, M et al. | 2015 | 62 | Hypospadias (Some cases were complicated with cryptorchidism or/and micropenis) | Copy number loss of the Y chromosome | 25605705 |
| 6 | Singh, Neetu et al. | 2018 | 143 | Hypospadias | 7q34 deletion | 30078147 |
| 7 | Scott, Carter H et al. | 2022 | 32 | Isolated and syndromic hypospadias (The phenotype is unspecified) | Investigated overlapping recurrent CNVs to identify 75 smallest regions of overlap (SROs) on 18 chromosomes. | 35457073 |
