# Supplementary material for: Novel evidence of CNV deletion in KCTD13 related to the severity of isolated hypospadias in Chinese population
Source: Front Pediatr. 2024 Sep 10;12:1409264. doi: 10.3389/fped.2024.1409264 (PMC11420791; doi:10.3389/fped.2024.1409264)
Supplement: Supplementary file 2 [file Table2.pdf]

Sample_id	Sex	Hypospadias classification	*KCTD13* copy number
S2024001	Male	Proximal	2
S2024002	Male	Proximal	2
S2024003	Male	Proximal	2
S2024004	Male	Proximal	2
S2024005	Male	Proximal	2
S2024006	Male	Proximal	1
S2024007	Male	Proximal	2
S2024008	Male	Proximal	2
S2024009	Male	Proximal	2
S2024010	Male	Proximal	2
S2024011	Male	Proximal	2
S2024012	Male	Proximal	2
S2024013	Male	Proximal	2
S2024014	Male	Proximal	2
S2024015	Male	Proximal	2
S2024016	Male	Proximal	1
S2024017	Male	Proximal	1
S2024018	Male	Proximal	2
S2024019	Male	Proximal	2
S2024020	Male	Proximal	1
S2024021	Male	Proximal	2
S2024022	Male	Proximal	2
S2024023	Male	Proximal	1
S2024024	Male	Proximal	2
S2024025	Male	Proximal	1
S2024026	Male	Proximal	1
S2024027	Male	Proximal	2
S2024028	Male	Proximal	2
S2024029	Male	Proximal	2
S2024030	Male	Proximal	2
S2024031	Male	Proximal	1
S2024032	Male	Proximal	2
S2024033	Male	Proximal	2
S2024034	Male	Proximal	2
S2024035	Male	Proximal	2
S2024036	Male	Proximal	2
S2024037	Male	Proximal	2
S2024038	Male	Proximal	2
S2024039	Male	Proximal	2
S2024040	Male	Proximal	2
S2024041	Male	Proximal	2
S2024042	Male	Proximal	2
S2024043	Male	Proximal	2
S2024044	Male	Proximal	2
S2024045	Male	Proximal	2
S2024046	Male	Proximal	2
S2024047	Male	Proximal	2

S2024048	Male	Proximal	2
S2024049	Male	Proximal	2
S2024050	Male	Proximal	2
S2024051	Male	Proximal	2
S2024052	Male	Proximal	2
S2024053	Male	Proximal	2
S2024054	Male	Proximal	2
S2024055	Male	Proximal	2
S2024056	Male	Proximal	1
S2024057	Male	Proximal	1
S2024058	Male	Proximal	2
S2024059	Male	Proximal	2
S2024060	Male	Proximal	2
S2024061	Male	Proximal	1
S2024062	Male	Proximal	1
S2024063	Male	Proximal	2
S2024064	Male	Proximal	2
S2024065	Male	Proximal	1
S2024066	Male	Proximal	1
S2024067	Male	Proximal	1
S2024068	Male	Proximal	1
S2024069	Male	Proximal	1
S2024070	Male	Proximal	1
S2024071	Male	Proximal	2
S2024072	Male	Proximal	2
S2024073	Male	Proximal	2
S2024074	Male	Proximal	1
S2024075	Male	Proximal	2
S2024076	Male	Proximal	2
S2024077	Male	Proximal	2
S2024078	Male	Proximal	2
S2024079	Male	Proximal	2
S2024080	Male	Proximal	2
S2024081	Male	Proximal	2
S2024082	Male	Proximal	1
S2024083	Male	Proximal	2
S2024084	Male	Proximal	2
S2024085	Male	Proximal	2
S2024086	Male	Proximal	1
S2024087	Male	Proximal	2
S2024088	Male	Proximal	2
S2024089	Male	Proximal	2
S2024090	Male	Proximal	2
S2024091	Male	Proximal	2
S2024092	Male	Proximal	2
S2024093	Male	Proximal	2
S2024094	Male	Proximal	2
S2024095	Male	Proximal	2

S2024096	Male	Proximal	2
S2024097	Male	Proximal	2
S2024098	Male	Proximal	2
S2024099	Male	Proximal	2
S2024100	Male	Proximal	2
S2024101	Male	Proximal	2
S2024102	Male	Proximal	2
S2024103	Male	Proximal	2
S2024104	Male	Proximal	2
S2024105	Male	Proximal	1
S2024106	Male	Proximal	2
S2024107	Male	Proximal	2
S2024108	Male	Midshaft	2
S2024109	Male	Midshaft	2
S2024110	Male	Midshaft	2
S2024111	Male	Midshaft	2
S2024112	Male	Midshaft	2
S2024113	Male	Midshaft	2
S2024114	Male	Midshaft	2
S2024115	Male	Midshaft	2
S2024116	Male	Midshaft	2
S2024117	Male	Midshaft	2
S2024118	Male	Midshaft	2
S2024119	Male	Midshaft	2
S2024120	Male	Midshaft	2
S2024121	Male	Midshaft	2
S2024122	Male	Midshaft	2
S2024123	Male	Midshaft	2
S2024124	Male	Midshaft	2
S2024125	Male	Midshaft	2
S2024126	Male	Midshaft	2
S2024127	Male	Midshaft	1
S2024128	Male	Midshaft	2
S2024129	Male	Midshaft	2
S2024130	Male	Midshaft	2
S2024131	Male	Midshaft	2
S2024132	Male	Midshaft	2
S2024133	Male	Midshaft	2
S2024134	Male	Midshaft	2
S2024135	Male	Midshaft	2
S2024136	Male	Midshaft	2
S2024137	Male	Midshaft	2
S2024138	Male	Midshaft	2
S2024139	Male	Midshaft	2
S2024140	Male	Midshaft	2
S2024141	Male	Midshaft	2
S2024142	Male	Midshaft	2
S2024143	Male	Midshaft	2

S2024144	Male	Midshaft	1
S2024145	Male	Midshaft	2
S2024146	Male	Midshaft	2
S2024147	Male	Midshaft	2
S2024148	Male	Midshaft	2
S2024149	Male	Midshaft	2
S2024150	Male	Midshaft	2
S2024151	Male	Midshaft	2
S2024152	Male	Midshaft	2
S2024153	Male	Midshaft	2
S2024154	Male	Midshaft	2
S2024155	Male	Midshaft	2
S2024156	Male	Midshaft	2
S2024157	Male	Midshaft	2
S2024158	Male	Midshaft	2
S2024159	Male	Midshaft	2
S2024160	Male	Midshaft	2
S2024161	Male	Midshaft	2
S2024162	Male	Midshaft	1
S2024163	Male	Midshaft	2
S2024164	Male	Midshaft	2
S2024165	Male	Midshaft	2
S2024166	Male	Midshaft	2
S2024167	Male	Midshaft	2
S2024168	Male	Midshaft	2
S2024169	Male	Midshaft	1
S2024170	Male	Midshaft	2
S2024171	Male	Midshaft	2
S2024172	Male	Midshaft	1
S2024173	Male	Midshaft	1
S2024174	Male	Midshaft	1
S2024175	Male	Midshaft	2
S2024176	Male	Midshaft	2
S2024177	Male	Midshaft	1
S2024178	Male	Midshaft	1
S2024179	Male	Midshaft	1
S2024180	Male	Midshaft	1
S2024181	Male	Midshaft	2
S2024182	Male	Midshaft	2
S2024183	Male	Midshaft	2
S2024184	Male	Midshaft	2
S2024185	Male	Midshaft	2
S2024186	Male	Midshaft	2
S2024187	Male	Midshaft	2
S2024188	Male	Midshaft	2
S2024189	Male	Midshaft	2
S2024190	Male	Midshaft	2
S2024191	Male	Midshaft	2

S2024192	Male	Midshaft	2
S2024193	Male	Midshaft	2
S2024194	Male	Midshaft	2
S2024195	Male	Midshaft	2
S2024196	Male	Midshaft	1
S2024197	Male	Midshaft	1
S2024198	Male	Midshaft	2
S2024199	Male	Midshaft	2
S2024200	Male	Midshaft	2
S2024201	Male	Midshaft	2
S2024202	Male	Midshaft	2
S2024203	Male	Midshaft	2
S2024204	Male	Midshaft	2
S2024205	Male	Midshaft	2
S2024206	Male	Midshaft	2
S2024207	Male	Midshaft	2
S2024208	Male	Midshaft	2
S2024209	Male	Midshaft	2
S2024210	Male	Midshaft	2
S2024211	Male	Midshaft	2
S2024212	Male	Midshaft	1
S2024213	Male	Midshaft	2
S2024214	Male	Midshaft	2
S2024215	Male	Midshaft	2
S2024216	Male	Midshaft	2
S2024217	Male	Midshaft	2
S2024218	Male	Midshaft	2
S2024219	Male	Midshaft	2
S2024220	Male	Midshaft	2
S2024221	Male	Distal	2
S2024222	Male	Distal	2
S2024223	Male	Distal	2
S2024224	Male	Distal	2
S2024225	Male	Distal	2
S2024226	Male	Distal	2
S2024227	Male	Distal	2
S2024228	Male	Distal	2
S2024229	Male	Distal	2
S2024230	Male	Distal	2
S2024231	Male	Distal	2
S2024232	Male	Distal	2
S2024233	Male	Distal	2
S2024234	Male	Distal	2
S2024235	Male	Distal	2
S2024236	Male	Distal	2
S2024237	Male	Distal	2
S2024238	Male	Distal	2
S2024239	Male	Distal	2

S2024240	Male	Distal	2
S2024241	Male	Distal	2
S2024242	Male	Distal	2
S2024243	Male	Distal	2
S2024244	Male	Distal	2
S2024245	Male	Distal	2
S2024246	Male	Distal	2
S2024247	Male	Distal	2
S2024248	Male	Distal	2
S2024249	Male	Distal	2
S2024250	Male	Distal	2
S2024251	Male	Distal	2
S2024252	Male	Distal	2
S2024253	Male	Distal	2
S2024254	Male	Distal	2
S2024255	Male	Distal	2
S2024256	Male	Distal	2
S2024257	Male	Distal	2
S2024258	Male	Distal	2
S2024259	Male	Distal	2
S2024260	Male	Distal	2
S2024261	Male	Distal	2
S2024262	Male	Distal	2
S2024263	Male	Distal	2
S2024264	Male	Distal	2
S2024265	Male	Distal	2
S2024266	Male	Distal	2
S2024267	Male	Distal	2
S2024268	Male	Distal	2
S2024269	Male	Distal	2
S2024270	Male	Distal	2
S2024271	Male	Distal	2
S2024272	Male	Distal	2
S2024273	Male	Distal	2
S2024274	Male	Distal	2
S2024275	Male	Distal	2
S2024276	Male	Distal	2
S2024277	Male	Distal	2
S2024278	Male	Distal	2
S2024279	Male	Distal	2
S2024280	Male	Distal	2
S2024281	Male	Distal	2
S2024282	Male	Distal	2
S2024283	Male	Distal	2
S2024284	Male	Distal	2
S2024285	Male	Distal	2
S2024286	Male	Distal	2
S2024287	Male	Distal	2

S2024288	Male	Distal	2
S2024289	Male	Distal	2
S2024290	Male	Distal	2
S2024291	Male	Distal	2
S2024292	Male	Distal	2
S2024293	Male	Distal	2
S2024294	Male	Distal	2
S2024295	Male	Distal	2
S2024296	Male	Distal	2
S2024297	Male	Distal	2
S2024298	Male	Distal	2
S2024299	Male	Distal	2
S2024300	Male	Distal	2
S2024301	Male	Distal	2
S2024302	Male	Distal	2
S2024303	Male	Distal	2
S2024304	Male	Distal	2
S2024305	Male	Distal	2
S2024306	Male	Distal	2
S2024307	Male	Distal	2
S2024308	Male	Distal	2
S2024309	Male	Distal	2
S2024310	Male	Distal	2
S2024311	Male	Distal	2
S2024312	Male	Distal	2
S2024313	Male	Distal	2
S2024314	Male	Distal	2
S2024315	Male	Distal	2
S2024316	Male	Distal	2
S2024317	Male	Distal	2
S2024318	Male	Distal	2
S2024319	Male	Distal	2
S2024320	Male	Distal	2
S2024321	Male	Distal	2
S2024322	Male	Distal	2
S2024323	Male	Distal	2
S2024324	Male	Distal	2
S2024325	Male	Distal	2
S2024326	Male	Distal	2
S2024327	Male	Distal	2
S2024328	Male	Distal	2
S2024329	Male	Distal	2
S2024330	Male	Distal	2
S2024331	Male	Distal	2
S2024332	Male	Distal	2
S2024333	Male	Distal	2
S2024334	Male	Distal	2
S2024335	Male	Distal	2

S2024336	Male	Distal	2
S2024337	Male	Distal	2
S2024338	Male	Distal	2
S2024339	Male	Distal	2
S2024340	Male	Distal	2
S2024341	Male	Distal	2
S2024342	Male	Distal	2
S2024343	Male	Distal	2
S2024344	Male	Distal	2
S2024345	Male	Distal	2
S2024346	Male	Distal	2
S2024347	Male	Distal	1
S2024348	Male	Distal	1
S2024349	Male	Distal	1
